# Time- and Gender-Dependent Alterations in Mice during the Aging Process

**DOI:** 10.3390/ijms241612790

**Published:** 2023-08-14

**Authors:** Jing Jin, Xiaoquan Yang, Hui Gong, Xiangning Li

**Affiliations:** 1 Britton Chance Center for Biomedical Photonics, Wuhan National Laboratory for Optoelectronics, MoE Key Laboratory for Biomedical Photonics, Huazhong University of Science and Technology, Wuhan 430074, Chinahuigong@mail.hust.edu.cn (H.G.); 2 Research Unit of Multimodal Cross Scale Neural Signal Detection and Imaging, HUST-Suzhou Institute for Brainsmatics, JITRI, Chinese Academy of Medical Sciences, Suzhou 215004, China; 3 Key Laboratory of Biomedical Engineering of Hainan Province, School of Biomedical Engineering, Hainan University, Haikou 570228, China

**Keywords:** aging, p21, distribution and production, gender factor, kidney, adrenal gland, C57

## Abstract

Compared to young people and adults, there are differences in the ability of elderly people to resist diseases or injuries, with some noticeable features being gender-dependent. However, gender differences in age-related viscera alterations are not clear. To evaluate a potential possibility of gender differences during the natural aging process, we used three age groups to investigate the impact on spleens, kidneys, and adrenal glands. The immunofluorescence results showed that male-specific p21 proteins were concentrated in the renal tubule epithelial cells of the kidney. Histological staining revealed an increase in the frequencies of fat vacuoles located in the renal tubule epithelial cells of the cortex, under the renal capsule in the kidneys of male mice with age. In female mice, we found that the width of the globular zone in the adrenal gland cortex was unchanged with age. On the contrary, the male displayed a reduction in width. Compared to females, the content of epinephrine in adrenal gland tissue according to ELISA analysis was higher in adults, and a greater decline was observed in aged males particularly. These data confirmed the age-dependent differences between female and male mice; therefore, gender should be considered one of the major factors for personalized treatment in clinical diagnosis and treatment.

## 1. Introduction

As the aging population continues to increase, diseases related to aging are receiving more attention. Over the years, many gender differences have been found to be related to susceptibility, progression, drug effects, and outcomes of some diseases, such as diabetes, heart disease, hypertension, and lung cancer [1,2,3,4,5,6]. Epidemiological studies reported that chronic kidney diseases in older men progress more rapidly than in older women [7], suggesting that there are gender differences in many aspects of the aging process [8]. Moreover, considering tissue specificity and common gender differences in epidemiology, individualized diagnosis and treatment in clinical practice may have a positive impact on patient prognosis. Nevertheless, existing research about the gender and tissue specificity of aging is still limited, and analyzing the features and molecular determinants of aging could help further elucidate the relevant mechanisms.

Increasing numbers of senescence cells in various organs were the main hallmarks of aging [9]. Several proteins have been identified to generate and maintain senescence, especially senescence-associated-β-galactosidase (SA-β-gal) [10], one of the most commonly used markers of cell senescence, as well as cyclin-dependent kinase inhibitor 1 (CDK1A), also known as p21 [9]. Cell cyclin regulator p16^ink4a^, an inhibitor of cyclin-dependent kinase 4 (CDK4), is strongly associated with aging in mice and rats [11]. Furthermore, an age-dependent raise in the expression of interleukin-6 (IL-6) in the spleen of aged mice [12], and p21 and p16 in zona glomerulosa (ZG) of the adrenal gland [13] highlights that the type of tissues and organs could affect the expression in terms of distribution and quantity of age-related proteins during the aging process. In addition to protein markers, morphological changes are also regarded as biomarkers, such as lipofuscin deposited in the proximal renal tubules of aging F344 rats [11], indicating that the aging extent of the renal tubules was higher than that of glomeruli. Nevertheless, studies investigating gender- and tissue-specific expression of these markers were still lacking.

Biochemical research explores the molecular basis of aging using various experimental animal models [14]. Laboratory mice are the most commonly studied mammal among animal models due to the similar pathophysiology to human disease and short lifespan [15,16,17]. Thus, the naturally aging mice could more faithfully recapitulate the features of human aging and associated diseases. However, there was a lack of comprehensive results on the age-related differences between genders. Thus, to investigate the influence of gender on age-related markers, we examined the expression of SA-β-gal, p21, and p16, and compared the morphology and protein secretion levels in the spleen, kidney, and adrenal gland of C57BL/6J mice at different life stages.

## 2. Results

### 2.1. Gender-Specific Differences in Senescence Cell Localization during Natural Aging

**Figure 1:**
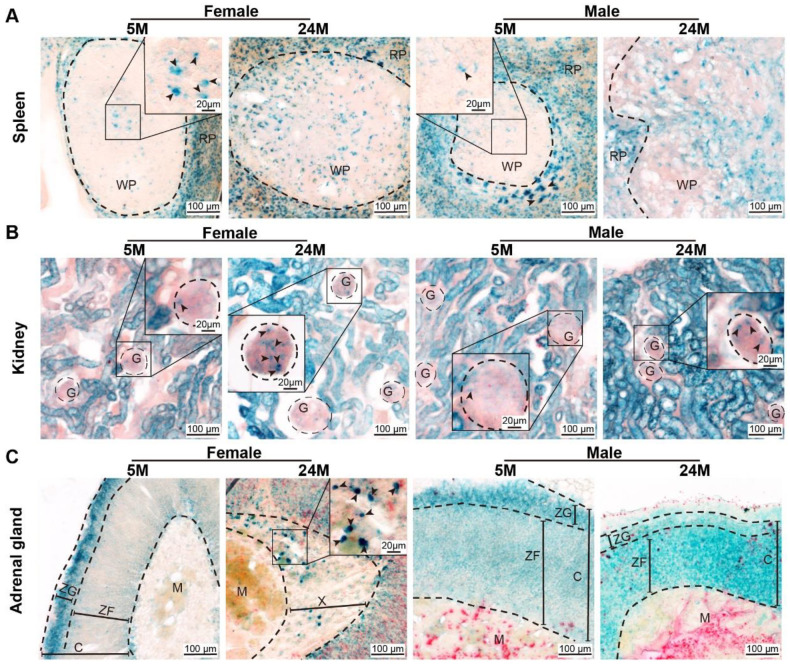
Age-dependent senescent cells. (**A**) Representative images of SA-β-gal expression in female and male mouse spleens. Note that the positive cells appeared in most of the red pulp (RP) and the center of white pulp (WP) initially, and then spread to the whole white pulp. Arrowheads indicate SA-β-gal-positive cells. (**B**) SA-β-gal expression in female and male kidneys revealed that the positive cells appeared in the renal tubules at the early time and were then expressed in the glomerulus (G), as shown by representative micrographs. Arrowheads indicate SA-β-gal-positive cells. (**C**) Representative images of SA-β-gal expression in female and male adrenal glands showed that the positive cells appeared in the zona glomerulosa (ZG) at an early time and then diffused through the whole cortex. Arrowheads indicated SA-β-gal-positive cells. ZF, zona fasciculata. C, the cortex of the adrenal gland. M, the medulla of the adrenal gland. X, the X-band of the adrenal gland.

To identify whether aging occurred in the organs of old mice, including the spleen, kidney, and adrenal gland, of both genders at different ages, including adults (3–5 months old), middle age (11–14 months old), and aged (>24 months old), we performed SA-β-gal staining to detect the senescent cells and the preferred/most recurrent region. The results showed that the red pulp in the spleen aggregated SA-β-gal-positive cells earlier and more frequently than white pulp (Supplementary Figure S1), and those cells first accumulated in a small amount in the white pulp center, then increased and gradually spread outward with age, eventually diffusing throughout the white pulp (Figure 1A). The kidney of mice began to senesce from the cortex (Supplementary Figure S1). SA-β-gal-positive cells were concentrated in renal tubules at first and then in the glomerulus with increased with age [11] (Figure 1B). The fact that the aging speed of renal tubules and glomeruli was different probably meant that the aging speed of renal tubules was slightly faster than that of glomeruli. Similarly, the adrenal gland cortex of mouse senesced earlier than the medulla (Supplementary Figure S1). In particular, ZG was the first area in our results and then spread in the zona reticularis (ZR), which could be named the X-band in mice (Figure 1C). There were few SA-β-gal-positive cells in the medulla, even in 24-month-old mice (Figure 1C).

**Figure 2:**
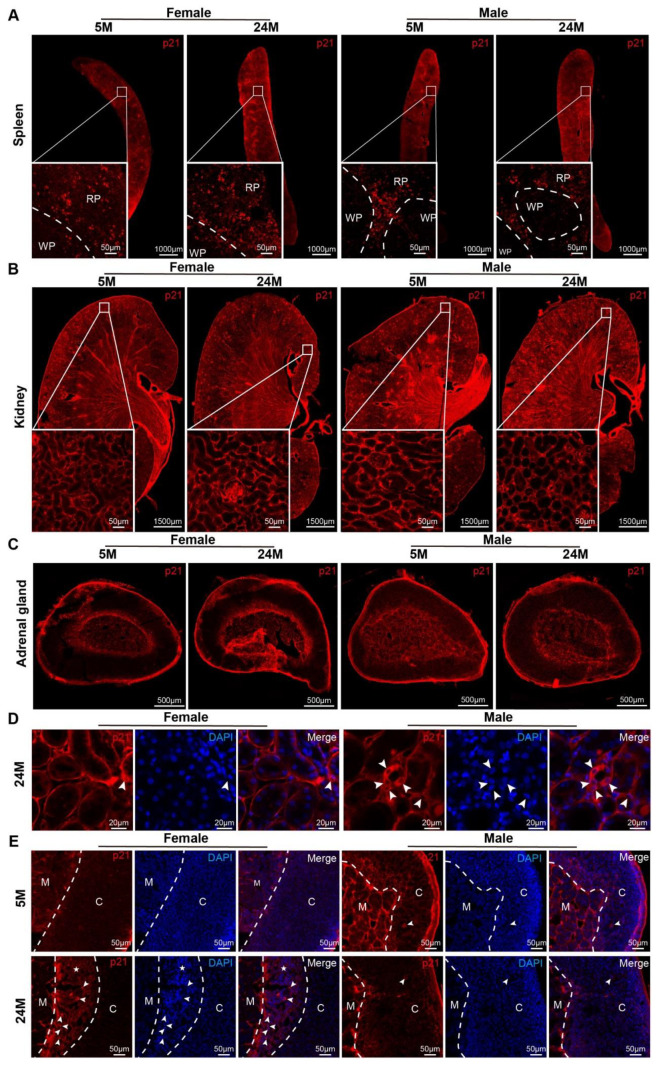
The expression and distribution of p21 in mice. (**A**–**C**) Representative photographs in the distribution of the p21 protein in mouse (**A**) spleens, (**B**) kidneys with zoomed-in views of boxed regions, and (**C**) adrenal glands. RP, red pulp. WP, white pulp. (**D**) Microscopy analysis of p21^+^ cell localization in the kidneys of 24-month-old male and female mice. In females, p21 was expressed in interstitial cells (white arrowhead), whereas males had it expressed in renal tubular epithelial cells (white arrowhead). (**E**) Representative images of p21 expression in adrenal glands in 5- or 24-month-old mice. In females, p21 was expressed in the X-band cells (white arrowhead), while in males, this occurred in ZF cells (white arrowhead). C, the cortex of the adrenal gland. M, the medulla of the adrenal gland.

There are many types of cells or tissues that constitute the cortex. We investigated whether all cortical cells would senesce together or in a certain order. P21 was detected via immunofluorescence staining in the tissues of aged mice [18,19]. The results displayed that p21-positive production was mainly expressed in the interstitial cells within the spleen (Figure 2A), kidney (Figure 2B), and adrenal gland (Figure 2C) in 5-month-old mice.

While the senescent cells were dramatically increased in both genders, there were significant differences in the distribution within the kidney and adrenal gland in males compared to females at 24 months of age. In the kidneys of aged female mice, p21 was found in the interstitium and renal parenchyma cells and tubular epithelial cells (Figure 2C). In the aged male mice, p21 was not only found in the interstitium and renal parenchyma cells, but also in tubular epithelial cells (Figure 2D).

With the increase in age, p21 gradually appeared in the parenchymal cell bundle of the cortex (Figure 2E). Particularly in females, there was obvious proliferation of X-band fibers (Figure 2E), which was consistent with the X-band aggregation of p21 in aged female mice. Male mice, in contrast, had this aggregated in cortical cells (Figure 2E). Unexpectedly, expression of the p16 protein in the spleen was not observed in our study, neither in the kidney nor in the adrenal gland (Supplementary Figure S2).

### 2.2. Gender Differences in Morphological and Histological Alteration

**Figure 3:**
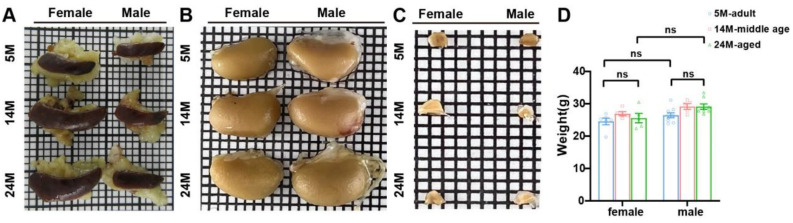
Age-dependent changes in spleen and kidney size. (**A**) Gross spleen, which was flat and oval. Aged mice had a bigger size than the adults. (**B**) Gross kidney. The kidney size of aged mice was larger than adult mice. (**C**) The adrenal gland was triangular and conical in appearance. (**D**) There was no difference in the body weight between aged mice and adult mice (*N* = 16 each group). ns, not significant. Data were expressed as the mean ± SEM and analyzed via a two-tailed unpaired Student’s *t*-test.

To examine the morphology changes with age, we performed routine staining via HE, PAS, and Masson. The results in 5-, 14-, and 24-month-old mice showed that the size of the spleen (Figure 3A) and kidney (Figure 3B) increased prominently with age, but the adrenal gland did not (Figure 3C). However, there was no obvious distinction in the weight of both genders (Figure 3D). Altogether, the results displayed an age-associated increase in spleen and kidney size.

Then, we investigated which microstructure had changed and which would be constant during the aging process. For this purpose, we performed histological staining analysis. The sections of the spleen showed that, compared to 5-month-old mice, the areas of the fibrotic region were larger, the boundary between the red and white pulp was blurred, the marginal zone formed a discontinuous boundary (Supplementary Figure S3A), and the number of macrophages in the marginal zone increased (Supplementary Figure S3B) in 24-month-old mice.

**Figure 4:**
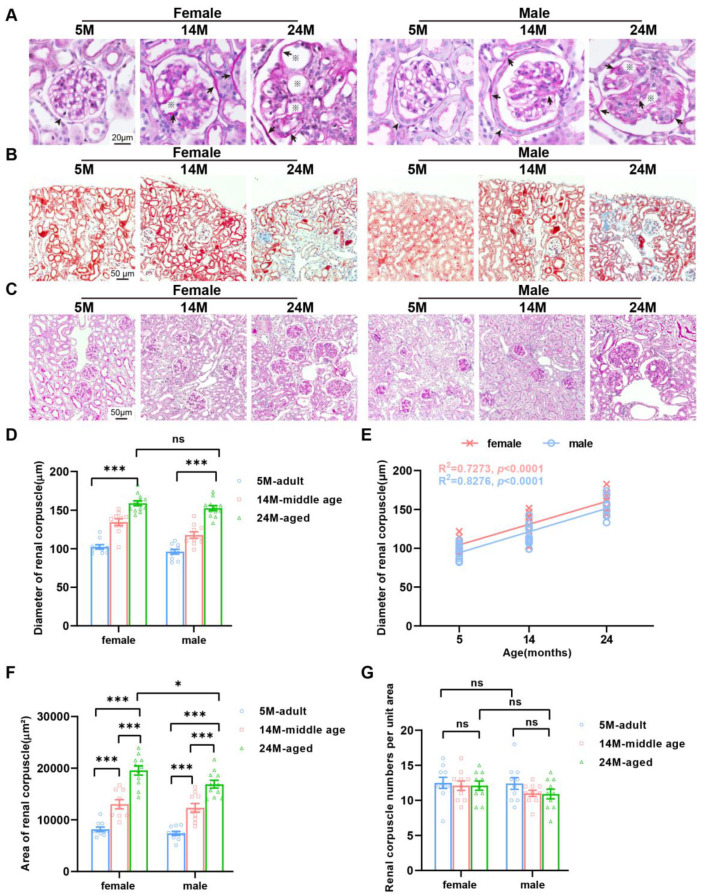
Gender-dependent changes in the glomerular diameter of an aging kidney. (**A**) Tissues processed for PAS staining showed that parietal epithelium cells in females were flat (black arrowhead), while they were single-layer cubic (black arrowhead) in males and had a thickened basement membrane (black arrow) and an enlarged capillary cavity (※) in both aged female and male mice. (**B**) Masson trichrome staining demonstrated increased renal fibrosis in mice of both genders. G, glomerulus. PT, proximal tubules. (**C**) Representative photomicrographs of PAS staining on sections of the mouse kidney. (**D**) Quantification of the average diameter of renal corpuscles. (**E**) Linear regression analyses showed that the renal corpuscle diameter was positively correlated with the female and male age (*N* = 16 each group). The square of Spearman’s correlation coefficient (*R*) and *p* values are shown. Data were statistically analyzed via linear regression (**F**,**G**). Graph represents (**F**) the average area and (**G**) number of renal corpuscles. *N* = 16 each group. * *p* < 0.05, *** *p* < 0.001. ns, not significant. Data are expressed as the mean ± SEM, a one-way ANOVA with Tukey’s test.

By employing the PAS staining method, it was observed that the cup-shaped upper skin sac with double walls was formed through the expansion and depression of the initial part of the renal tubule. In females, the parietal epithelium was flat, while it was single-layer cubic in males (Figure 4A). It was also found that the glomerular basement membrane of 5-month-old mice was noticeably thinner than that of 14- and 24-month-old mice (Figure 4A). This indicated that the glomerular basement membrane thickened with age. The glomerular capillaries of 24-month-old mice also showed severe dilation of the lumen (Figure 4A). Masson staining exhibited that the area of renal interstitial fibrosis in 24-month-old mice was more than that in 5-month-old mice, indicating that renal fibrosis was also aggravated with age (Figure 4B).

The glomerular diameter of 5-month-old mice was markedly smaller than that of 24-month-old mice (Figure 4C). Our calculation and statistics present that the increase in glomerular diameter of female mice was more obvious than that of male mice (Figure 4D,E). Further observation showed that the glomerular areas of 5-month-old mice were apparently smaller than those of 24-month-old mice (Figure 4F). However, the histological section statistics showed that there was no change in the number of glomeruli with age (Figure 4G). Combined with the above results, we speculated that with age, glomeruli in mice would become hypertrophied rather than atrophying or disappearing.

**Figure 5:**
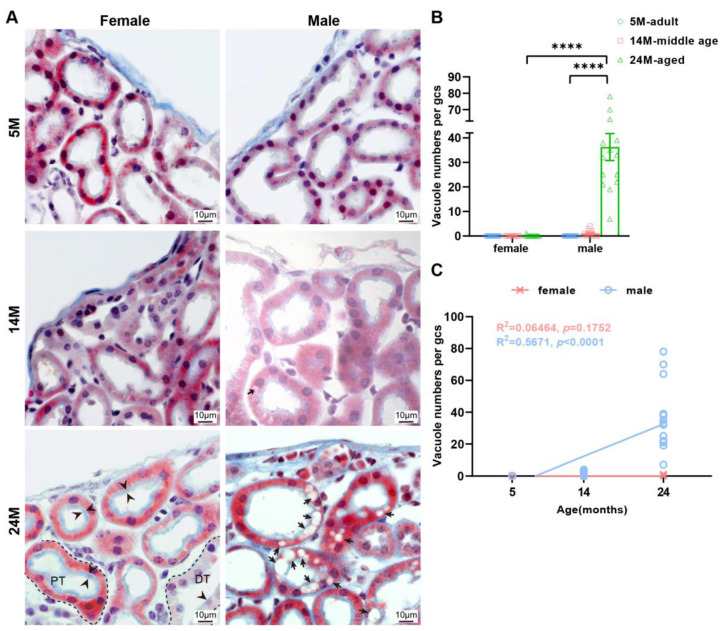
The tubular epithelial cells of male mice kidney are more vesicular compared to females. (**A**) Representative photos of renal tubules under renal capsule via Masson staining. Note the brush border on the free surface of the cells (black arrowhead) and vacuoles in the epithelial cells of renal tubules (black arrow) in the subcapsular cortex of male mice. PT, proximal tubules. DT, distal tubule. (**B**) Quantitative analysis of the results was shown. Data were shown as the mean ± SEM, a one-way ANOVA with Tukey’s test. **** *p* < 0.0001. (**C**) Linear regression analyses showed that the vacuole numbers had positively correlated with the male age but were uncorrelated with female age (*N* = 16 each group). The square of Spearman’s correlation coefficient (*R*) and *p* values are shown. Data were statistically analyzed via linear regression.

In 14-month-old male mice, only a small number of renal tubular epithelial cells in the renal subcapsular cortex contained vacuoles (Figure 5A), but the number increased significantly in the 24- month-old male mice (Figure 5B). At the same time, this phenomenon was not observed in 5-month-old male mice and female mice of all ages. This revealed that the appearance of vacuoles in the epithelial cells of renal tubules in the renal subcapsular cortex was a male-specific (Figure 5C), age-dependent change.

**Figure 6:**
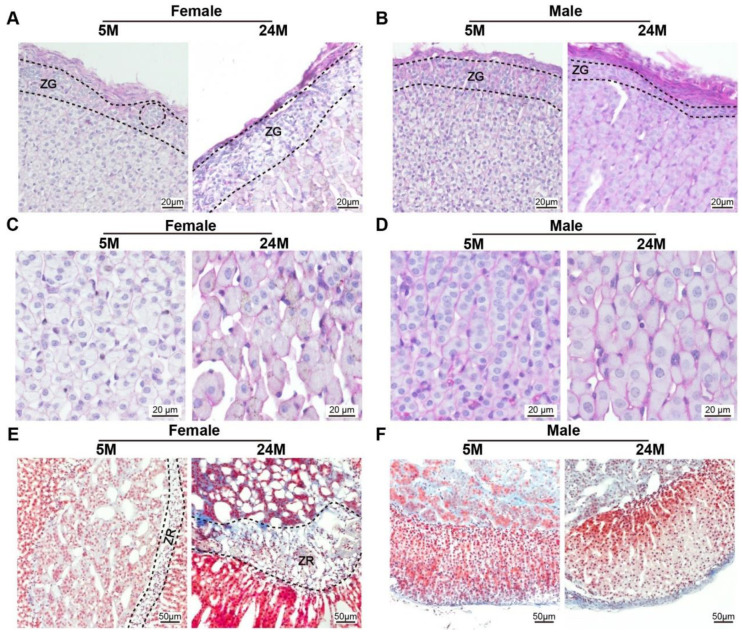
The glomerular zone of the male mouse adrenal gland cortex was thinner compared to females (**A**,**B**). Representative photomicrographs of PAS staining on the sections of the mouse adrenal gland showed that (**A**) the width of the zona glomerulosa (ZG) in female mice adrenal gland becomes thicker, while (**B**) the male globular zone becomes thinner during the aging process. (**C**,**D**). Note that the size of the ZF cells was larger with age (**E**,**F**). Adrenal gland tissues subjected to Masson staining revealed that (**E**) the zone reticularis (ZR), known as the X-zone, became wider with age in female mice, (**F**) but was absent in the lifetime of male mice.

There was no difference in the thickness of the glomerular zone in the adrenal cortex among female mice of any age (Figure 6A). However, the thickness of the glomerular zone of the adrenal cortex in 24-month-old male mice was substantially smaller than that in 5-month-old male mice (Figure 6B). The zona fasciculata cells of the adrenal cortex of 5-month-old mice was greater than those of the 24-month-old mice (Figure 6C,D). This reflected that the cell volume of the fascicular zone of the adrenal cortex increased with age in both genders. It was found that the width of the reticular zone in 24-month-old female mice was obviously greater due to lipofuscin deposition and fiber proliferation than that in 5-month-old female mice (Figure 6E,F).

### 2.3. Functional Changes in Protein Secretion during Natural Aging

**Figure 7:**
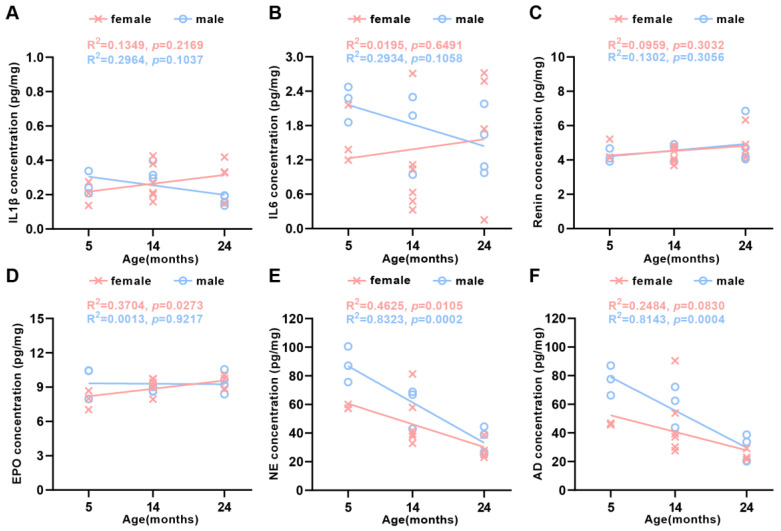
Production levels of protein secretion in the spleen, kidney, and adrenal gland in aging mice. Linear regression analyses showed that (**A**) IL-1β, (**B**) IL-6, and (**C**) renin were uncorrelated with the female and the male age. (**D**) EPO was positively correlated with female age, but uncorrelated with male age, (**E**) NE was inversely correlated with both female and male age, and (**F**) AD was uncorrelated with the female age, while it was inversely correlated with male age (*N* = 23 each group). The square of Spearman’s correlation coefficient (*R*) and *p* values are shown. Data were statistically analyzed via linear regression.

There was increasing evidence indicating that senescence contributed to the development and progression of body dysfunction [9]. Cells and tissues secreted specific proteins due to their different functions, while the corresponding protein levels indirectly reflected the functional state of the organization. To investigate the age-dependent effects on tissue function changes, the production levels of secreted proteins, including interleukin 1 beta (IL-1β) and IL-6, were detected by means of quantitative ELISA in different age groups, respectively. These were two of the main inflammatory factors produced by spleen immune cells [20]. Although IL-1β (Figure 7A) and IL-6 (Figure 7B) levels were higher in 24-month-old female mice than those in male ones, there was no significant difference. The levels of erythropoietin (EPO) and renin secreted by glomerular granulosa cells in the kidneys could indicate the status of renal function [21,22]. We noticed that renin levels were stable in 24-month-old and 5-month-old mice in both genders (Figure 7C). The finding that erythropoietin levels were a little higher in 24-month-old female mice and unchanged in males (Figure 7D) implied that there were significant main effects of age and gender on erythropoietin protein levels, with a significant interaction between age and gender in the kidney. Adrenal medullary chromaffin cells primarily secreted epinephrine and norepinephrine [23]. Norepinephrine levels were remarkable reduced with aging in male and female mice (Figure 7E). Similarly, epinephrine levels also declined in 24-month-old female and male mice (Figure 7F). As seen in the results, age significantly impacted epinephrine and norepinephrine in the adrenal gland. With respect to gender, the dramatically greater catecholamine in male than female mice adrenal gland expanded om previous knowledge, showing that the production levels of epinephrine and norepinephrine tended to increase in the adrenal glands of male mice.

## 3. Discussion

The structural and functional changes in aging cells are key features of aging organ function decline [9]. Gender is an important regulatory factor in disease development [24], but the role of gender difference in aging is yet to be explored. Hence, the aim of this study was to compare males and females in the distribution of senescent protein, the characteristics of histological alterations, and the level of main functional protein production during the natural aging process. We observed that during natural aging, there were a number of important differences between males and females. P21 tended to be expressed in male renal tubular epithelial cells and ZG, as well as in the female X-band. It is worth mentioning that vesicles also preferred to aggregate in male renal tubular epithelial cells. In addition, although adrenaline and norepinephrine continued decreasing with age, the production level of males was higher than that of females at the same age, and this advantage was maintained until the mice were 24 months old. Taken together, our data demonstrated that the aging process between different tissues and cells in organs was synchronous and the extent of aging in the same tissue and cell was also out of sync between males and females, providing a possibility that gender may be of a certain value in individualized regimens for aging and age-related diseases, and could potentially direct clinical treatment.

Cell senescence leads to irreversible cell cycle arrest, and the loss of ability to divide, grow, and repair, manifested by decreased oxidative phosphorylation, respiratory rate, and receptor proteins [25]. The upregulation and increase in lysosomal protein content led to the high activity of SA-β-gal, which is related to aging [10,26]. SA-β-gal increased in the epithelium with growth and aging, and their occurrence was significantly associated with each other [11]. Our findings were in line with these publications and expanded the boundary of the species used in this pattern. We discovered that the cortex displayed deeper SA-β-gal activity staining than the medulla, demonstrating that the relative extent of senescence from different tissue types in one organ was out of sync with age.

Several studies show that p21^CIP1/WAF1^ plays an important role in cell senescence and is associated with age [27]. Our findings support a statement that the structural changes caused by kidney aging may increase sensitivity to damage, so the elderly may be prone to nephrotoxicity [28,29]. We examined p21 via immunofluorescence in renal tubular epithelial cells at an old stage in male mice rather than age-matched females, highlighting that, compared with females, the senescence of male tubular epithelial cells occurred more rapidly, and that they might be more susceptible. Compared to previous studies [18], we identified cell types that expressed p21 and revealed more possible reasons for the expression of p21 in male kidneys. It has been reported that the accumulation of senescent cells in the aged kidney was related to the age-related decline in renal function [29,30,31]. The increased p21 expression in the kidney of allograft nephropathy patients might be responsible for the poor survival of transplants from elderly donors in clinical practice [32]. Regarding the adrenal gland, aged male mice aggregated p21 in cortical cells, in contrast to the X-band aggregation of p21 in aged female mice. We also noticed that the p21 was expressed the earliest in interstitial cells in the above-mentioned organs. These phenomena suggested that interstitial cells might be more susceptible to aging [33,34] or p21 might be a constitutive expression in interstitial cells that was not affected by time, location, or environment, and had no spatiotemporal specificity [35]. Briefly, on the one hand, this explained that the aging progress of the same cell type in the same organ varied between aged male and female mice. On the other hand, the difference in the aging cell location and relative degree of aging in tissues of mice with the natural aging might reflect different levels of genotoxic stress and/or different responses to stress. Cell cyclin regulator p16^ink4a^ is strongly associated with aging in mice and rats [9]. In contrast to earlier findings, however, no evidence of p16 was detected in our results, which may be related to the specificity of the antibody. However, recent reports support the idea that the p16 protein is rarely expressed in the spleen [18,19,36]. As a result, p16 might not be suitable for characterizing aging cells in the spleen or other organs of some mice stains [18,36].

Unlike human studies, where kidney aging caused atrophy, several studies have shown that the spleen decreased in size with age [37,38], the size of kidney [13] increased, and the adrenal gland size remained the same [23] in aged mice. Our findings support the above statement, meaning that different organs could have different size changes during aging. As previously described [39], the glomerular diameter enlarged without the number of glomeruli changing during aging. Our results were similar to those reported. Indeed, there is good reason to believe that the increase in kidney volume in mice was at least partly due to an increase in glomerular volume. We observed a vacuole-specific accumulation associated with the epithelial cells of renal tubules under the renal cortex of aged male mice. Comparing to the past findings in DBA/2Cr mice [40], we not only verified the existence of vacuoles in male C57 renal tubular epithelial cells, but also found the rhythmical time sequence, gender, and histiocyte types of these morphological changes in vacuoles. On the one hand, this might be related to the fact that the prevalence of chronic kidney disease in elderly men is much higher than in women in clinical practice. On the other hand, the existence of non-isometric vacuoles [41] is a common finding in ischemic injury, in addition to glomerulomegaly, thickening of the basement membrane, and capillary enlargement. Vascular endothelial cells gradually decrease during aging [42,43] and the decrease in renal function and blood flow during aging is closely related to the decrease in perivascular density [44,45]. Hence, vascular damage and/or ischemic injury might play an important role in kidney aging [46]. The adrenal glands of mice present androgynous tissue, size, and function [47,48,49,50], and ZG is the most senescent and undergoes a sudden decline in men [13]. Our results also identified this specificity of male ZG. The thickness of ZG changed with different trends, thinning in males, but being invariant in female mice.

IL-1β and IL-6 are considered participants in the tissue aging processes [20]. A decrease in the number of IL-1β- and IL-6-positive cells in the spleen of rats [37] and splenic stromal cells expressed higher levels of IL-6 [12]. Hence, it is reasonable to assume that a small number of positive cells produced more IL-1β and IL-6, ultimately manifested as a constant level. Considering the above possibilities, in the present study, it was reasonable to maintain the same level of IL-1β and IL-6. This may be an adaptive change made by the spleen to maintain a stable internal environment. Notably, some studies may discover opposite results due to the detection of elevated levels in the plasma [51,52]. Erythropoietin acts on nonhemopoietic cells in a cytoprotective manner in damaged tissues and organs, including the brain, kidney, cardiovasculature, and liver [53]. Therefore, the increase in erythropoietin in female kidneys may be a manifestation of antagonizing aging, causing lower levels of fibrosis, making them less susceptible to damage and less favored by diseases. The catecholamine synthesis was observed to be low in aged mice [23]. Our results were similar to comparable studies. Interestingly, the dysfunction of the adrenal gland to produce adrenaline occurred when the morphology of chromaffin cells was still normal. Alongside this, we identified that the production levels of catecholamine in male mice were significantly higher compared with those from age-matched females until 24 months of age. Additional studies are needed to clarify the mechanisms related to the reduced production levels of catecholamine in chromaffin cells with age and gender. In order to overcome the difficulties, it may be necessary to further investigate how nerves regulate the secretion of catecholamines in chromaffin cells.

## 4. Materials and Methods

### 4.1. Animal

Adult mice of the C57 strain were used in these experiments. In brief, the animals were sorted into three groups: adult (3–5 months, n = 24), middle age (11–14 months, n = 24), and aged (>24 months, n = 24). Mice were purchased from Viton Lihua (license no.: SCXK (Zhe) 2019-0001) and maintained in an SPF animal room (21 °C ± 1 °C). The mice were fed with free food and water, and the light was circulated for 12 h. All animal experiments were approved by Huazhong University of Science and Technology of Institutional Animal Care and Use Committee and adhered to the Huazhong University of Science and Technology of Health guidelines.

### 4.2. Reagent 

The sources of experimental reagents are shown in Table 1.

### 4.3. Antibody List

The usage details of antibodies are shown in Table 2.

### 4.4. Perfusion Fixation

Mice were deeply anesthetized using an intraperitoneal injection (of 2% chloral hydrate and 10% ethyl carbamate). The anesthetized mice were secured on a platform and the chest skin was cut to passively separate the subcutaneous tissue. Then, the ribs were cut, and the chest was fully exposed. The perfusion needle was inserted into the aorta from the tip of the heart and fixed. After opening the rib cage, a needle was inserted through the left ventricle of the mouse. Mice were perfused transcardially with 10 mL PBS (37 °C) and then 10 mL of ice-cold 4% paraformaldehyde using an electric pump at a 2 mL/min pump rate.

### 4.5. Sampling

After perfusion fixation, the spleen, kidney, and adrenal gland were collected and placed in 4% paraformaldehyde for storage at 4 °C overnight.

### 4.6. Frozen Section

The tissue was transferred to PBS solutions containing 20% sucrose to precipitate sugar, and then transferred to a 25% sucrose solution overnight after the tissue was immersed. The next day, the immersed tissue was transferred to 30% sucrose solutions. After sinking to the bottom, it was embedded with OCT glue, and sliced at −20 °C. The slice thickness was 20 μm.

### 4.7. Paraffin Section

The tissue was washed in 0.01 mol/L PBS for 6–8 h, and this was repeated 3 times. The rinsed tissues were infused with 70% alcohol for 4–6 h, 80% alcohol for 4–6 h, 95% alcohol for 4–6 h, 100% alcohol Ⅰ for 1.5 h, and 100% alcohol Ⅱ for 1.5 h, and then kept in 1-butanol overnight. After soaking in paraffin for 3 h, the tissue was embedded in the paraffin. The slice thickness was 3 μm.

### 4.8. Histology and Immunofluorescence

Mouse paraffin-embedded sections (3 µm thickness) fixed in 4% paraformaldehyde were subjected to a periodic acid–Schiff (PAS) stain, HE stain, and Masson stain according to the manufacturer’s procedures. The slides were imaged using an ECLIPSE Ni-E microscope (Nikon Corporation, Tokyo, Japan).

The frozen sections (20 µm thickness) were washed 3 times in 0.01 mol/L PBS for 5 min each time and then permeabilizated in 0.01 mol/L PBS with 0.3% Triton X-100 and 0.3% H_2_O_2_ at room temperature for 30 min. Then, the sections were blocked in 0.01 mol/L PBS with 3% BSA for 1 h. Antibodies were diluted in 0.01 mol/L PBS with 3% BSA and 0.3% Triton X-100, and the sections were incubated with the primary antibody overnight at 4 °C. The next day, after being washed with 0.01 mol/L PBS 3 times, for 5 min each time, the sections were incubated with the secondary antibody in the dark at room temperature for 1 h. Slices were washed with 0.01 mol/L PBS 3 times, for 5 min each time. In the end, the slices were incubated with DAPI or PI in the dark for 10 min and mounted in 0.01 mol/L PBS with 50% glycerol after washing. Images were acquired using an LSM710 confocal microscope (Carl Zeiss, Oberkochen, Germany).

### 4.9. Staining

The frozen sections (20 µm thickness) underwent SA-β-gal staining according to the guidelines of the manufacturer. SA-β-gal staining was used to test the β-galactosidase activity for the detection of senescence, and the sections were also imaged using an ECLIPSE Ni-E microscope.

### 4.10. Enzyme-Linked Immunosorbent Assay (ELISA)

The levels of IL-1β, IL-6, erythropoietin, renin, epinephrine, and norepinephrine produced in tissues were quantified using the double antibody sandwiched method, utilizing commercially available antibodies and according to the protocol provided by the supplier. IL-1β, IL-6, erythropoietin, renin, epinephrine, or norepinephrine bound to the HRP-labeled antibody to form an antibody–antigen enzyme-labeled antibody complex. After thorough washing, the substrate TMB was added for color development. TMB was converted into blue under HRP enzyme catalysis, and then turned yellow after stop solution addition. The OD values were detected at 450 nm. The concentrations of IL-1β, IL-6, erythropoietin, renin, epinephrine, and norepinephrine were determined based on a standard curve multiplied by the sample dilution factor.

### 4.11. Statistics

All in vitro experiments were performed at least 3 times. Numerical data are presented/expressed as the mean ± SEM. Statistical analyses were performed using SPSS version 24 (IBM Software, Armonk, NY, USA) and GraphPad Prism 8.0 (GraphPad Software, San Diego, CA, USA). The number of glomeruli was calculated per unit area in each file. Of the cross-sectional slides, 5–10 were randomly selected from each sample, with at least 5 files randomly selected from each slice. Data were analyzed using Student’s *t*-test or the Mann–Whitney U-test when normality could not be assumed. A two-way ANOVA was performed on data to assess the main effects of gender and age. If significant differences were found, the Bonferroni post hoc test was used to determine the source of the difference. Linear regression analysis was applied to test the possible relationships between the 2 parameters. *p* < 0.05 was considered statistically significant.

## 5. Conclusions

Our study focused on the evaluation of aging-associated alterations, combining functional changes, such as senescence markers, histological characteristics, and protein secretion. We determined the age-dependent and tissue-specific modulation of senescent cells during natural aging. Furthermore, the mouse spleen, kidney, and adrenal gland underwent both morphological and functional changes in response to an increase in age, significantly influenced by gender, which may potentially influence the rate and extent to which males and females age and develop age-related diseases.

## Figures and Tables

**Table 1:** Reagent List. The product number and manufacturer of the reagent were listed below.

Reagent Name	Catalog Number	Company
PBS	P3813	Sigma-Aldrich, St. Louis, MO, USA
Paraformaldehyde	158127	Sigma-Aldrich, St. Louis, MO, USA
Chloral hydrate	30037517	Sinopharm Chemical Reagent Co., Ltd., Shanghai, China
Ethyl carbamate	30191218	Sinopharm Chemical Reagent Co., Ltd., Shanghai, China
OCT	4583	SAKURA, Tokyo, Japan
PI	P21493	Thermo Fisher Scientific, Waltham, MA USA
DAPI	D1306	Thermo Fisher Scientific, Waltham, MA USA
Triton X-100	T9284	Sigma-Aldrich, St. Louis, MO, USA
Bovine serum albumin	A1933	Sigma-Aldrich, St. Louis, MO, USA
Ethanol	10009218	Sinopharm Chemical Reagent Co., Ltd., Shanghai, China
Butyl alcohol	10005218	Sinopharm Chemical Reagent Co., Ltd., Shanghai, China
Glycerol	10010618	Sinopharm Chemical Reagent Co., Ltd., Shanghai, China
Dimethylbenzene	10023418	Sinopharm Chemical Reagent Co., Ltd., Shanghai, China
β-Galactose staining kit	9860	Cell Signaling, Danvers, MA, USA
PAS staining kit	BH0003	POWERFUL BIOLOGY, Wuhan, China
Masson staining kit	BH0002	POWERFUL BIOLOGY, Wuhan, China
Noradrenaline ELISA Kit	JYM0577Mo	Colorful-Gene biological technology, Wuhan, China
Interleukin-6 ELISA Kit	JYM0012Mo	Colorful-Gene biological technology, Wuhan, China
Epinephrine ELISA Kit	JYM0287Mo	Colorful-Gene biological technology, Wuhan, China
Erythropoietin ELISA Kit	JYM0114Mo	Colorful-Gene biological technology, Wuhan, China
Renin ELISA Kit	JYM0269Mo	Colorful-Gene biological technology, Wuhan, China
Interleukin-1β ELISA Kit	JYM0531Mo	Colorful-Gene biological technology, Wuhan, China

**Table 2:** Antibody List. The product number, manufacturer, and dilition of antibody were shown.

Antigen	Cat#	Company	Dilution
Anti-p21 antibody	sc-6246	Santa Cruz Biotechnology, CA, USA	1:200
Anti-p16 antibody	ab28486	Abcam, Cambridge, UK	1:200
Goat Anti-Mouse-Alexa488	A-11029	Invitrogen, Waltham, MA, USA	1:800
Goat Anti-Rabbit-Alexa488	A-11034	Invitrogen, Waltham, MA, USA	1:800
Goat Anti-Mouse-Alexa594	A-11032	Invitrogen, Waltham, MA, USA	1:800
Goat Anti-Rabbit-Alexa594	A-11037	Invitrogen, Waltham, MA, USA	1:800

## Data Availability

The original contributions presented in the study are included in the article/supplementary files. Further inquiries can be directed to the corresponding authors.

## Supplementary Materials

The following supporting information can be downloaded at: https://www.mdpi.com/article/10.3390/ijms241612790/s1.
