# Supplementary material for: Time- and Gender-Dependent Alterations in Mice during the Aging Process
Source: Int J Mol Sci. 2023 Aug 14;24(16):12790. doi: 10.3390/ijms241612790 (PMC10454612; doi:10.3390/ijms241612790)
Supplement: Supplementary file 1 [file ijms-24-12790-s001.zip › ijms-2487619-supplementary.pdf]

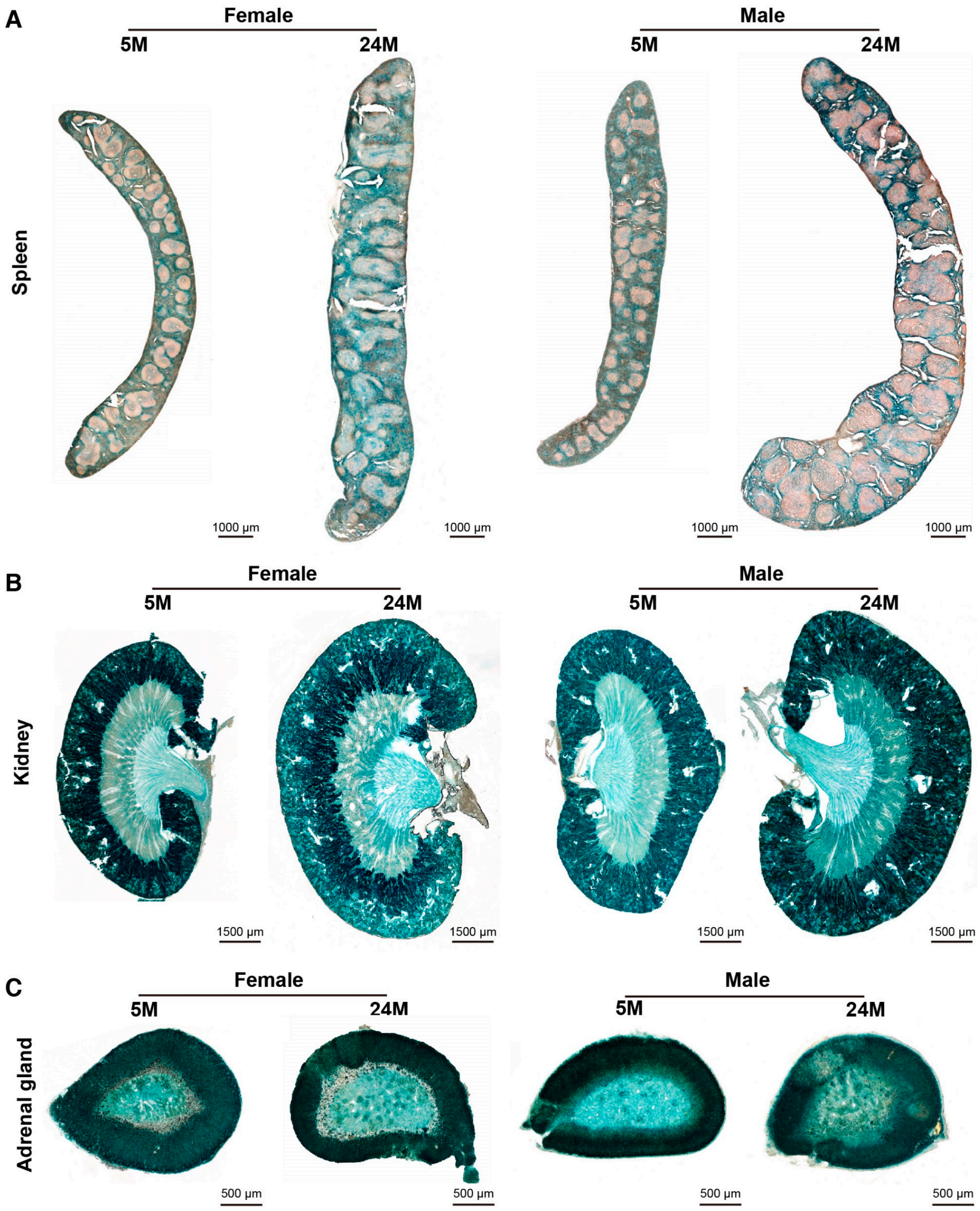

Scheme S1. Age-dependent senescent cells increased. (A) Representative images in distribution of SA-β-gal expression in female and male spleens at 5M or 24M. Note the staining degree of RP was heavier than that of WP. (B) Representative images in distribution of SA-β-gal expression in female and male kidneys at 5M or 24M. Note the staining degree of cortex was heavier than that of medulla. (C) Representative images in distribution of SA-β-gal expression in female and male adrenal glands at 5M or 24M. Note the staining degree of cortex was heavier than that of medulla. All experiments were repeated three times with similar results.

.

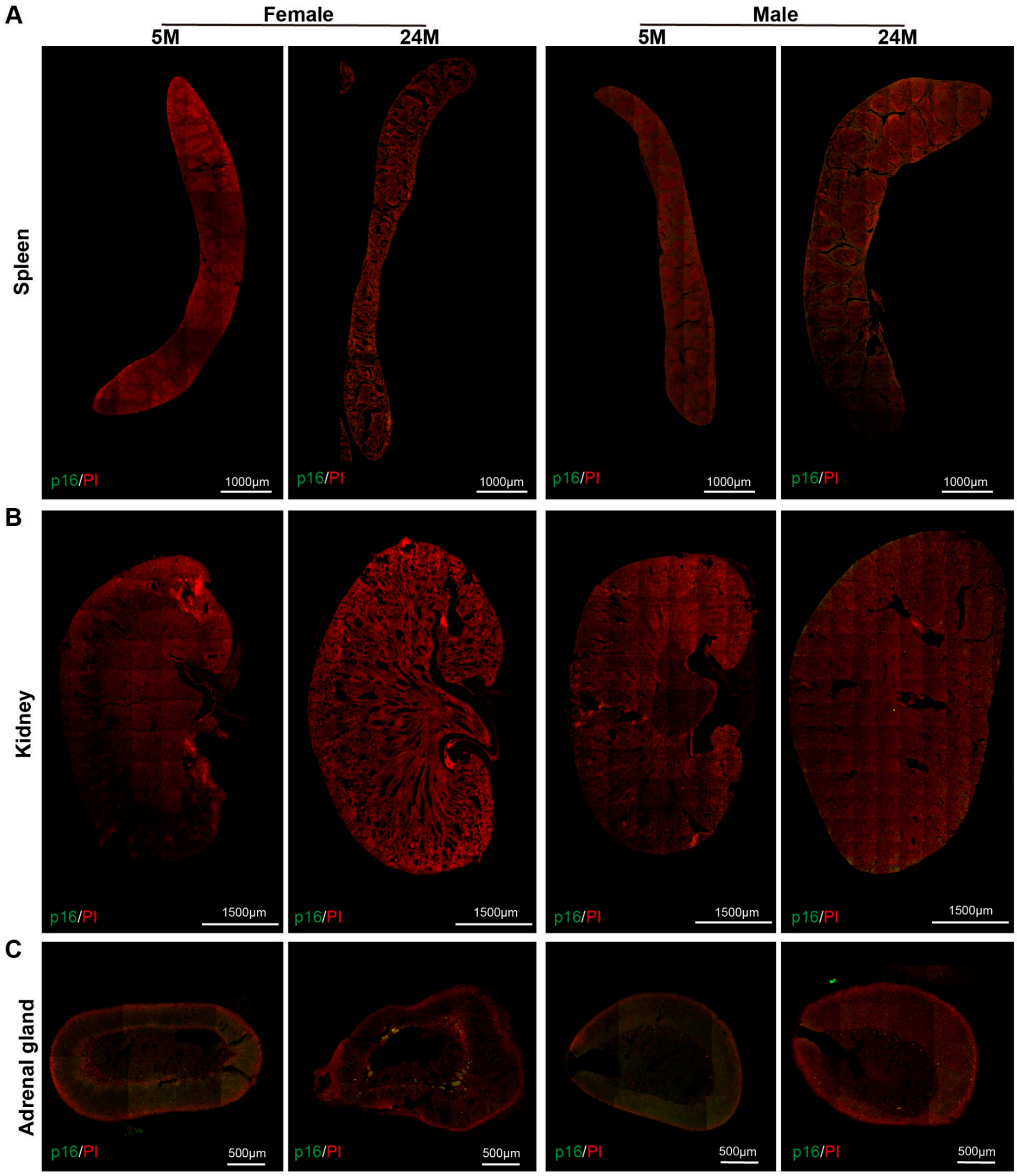

Scheme S2. The expression and distribution of p16 in mice. Few p16 protein expression could be seen.

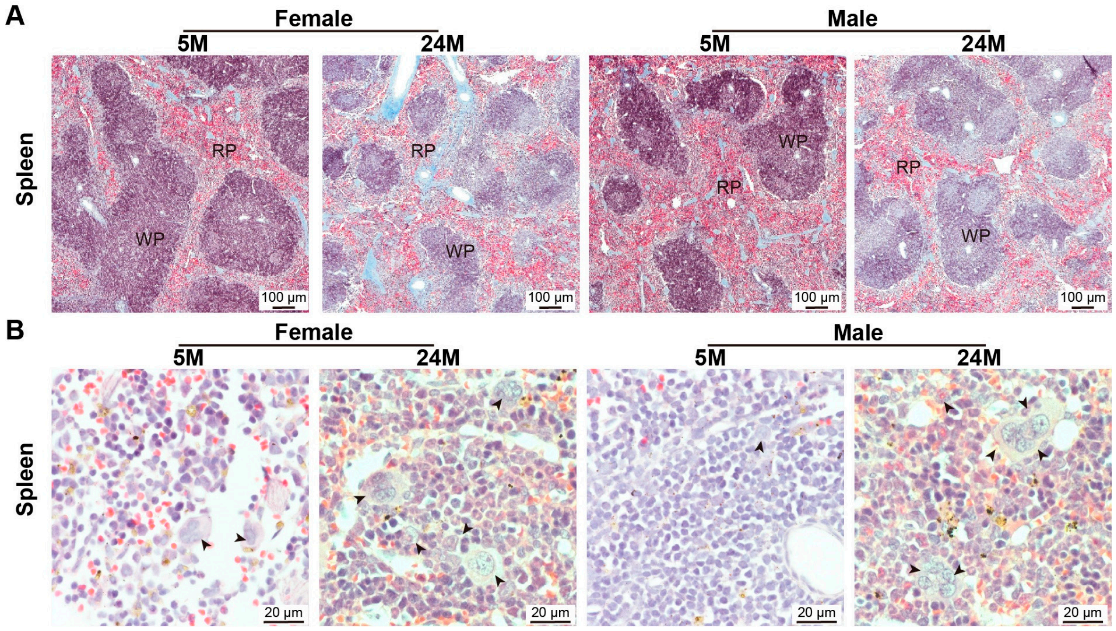

Scheme S3. There was no gender difference in age-dependent changes in morphological in spleen. (A) Representative photomicrographs of Masson staining on sections of mice spleen. Note the boundary between red pulp (RP) and white pulp (WP) became blurred in 24-month-old mice. (B) Representative photomicrographs of HE staining on sections of mice spleen. Note the size of macrophages (black arrowhead).
